# Long-term response with low-dose of apatinib combined with S-1 in pretreated patient with advanced squamous cell lung cancer: A case report

**DOI:** 10.1097/MD.0000000000024390

**Published:** 2021-02-26

**Authors:** Jianxin Chen, Junhui Wang, Yan Zou

**Affiliations:** a Department of Medical Oncology; b Department of Radiation Oncology, Quzhou People′s Hospital, Quzhou, Zhejiang, China.

**Keywords:** apatinib, case report, progressive free survival, S-1, squamous cell lung cancer

## Abstract

**Rationale:**

Squamous cell lung cancer is one of the major pathological types in patients with non-small cell lung cancer. Since treatment with angiogenic agents and target drugs in patients with advanced squamous cell lung cancer is not promising, there are limited strategies to improve the outcome in such patients. Herein, we report a pretreated patient with advanced squamous cell lung cancer, who received low-dose of apatinib combined with S-1 as salvage treatment, with good long-term response.

**Patient concerns:**

The patient complained of dry cough for one month without any relief by medication. Otherwise, she denied any other medical or family history.

**Diagnosis:**

According to the chest computed tomography, and pathologic findings from biopsy for lesion in lung, the patient was diagnosed with lung squamous cell lung cancer with enlargement of bilateral supraclavicular lymph nodes suggesting metastasis, staged as IIIb.

**Interventions:**

The patient received gemcitabine plus cisplatin as first line treatment, and gemcitabine as maintenance therapy. After progression, she received vinorelbine as second line treatment. After progression again, she received low-dose apatinib combined with S-1 as third line treatment.

**Outcomes:**

With the follow-up period from October 21, 2014, to April 6, 2019, there were 15 months, 9 months, and 24 months of progression-free survival time for first line (including maintenance therapy), second line, and third line treatment, respectively. The only adverse event was neutropenia at grade 2 (CTC AE) occurring during the maintenance treatment.

**Lessons:**

This case indicated that low-dose apatinib combined with S-1 might be effective and safe in selected pretreated patients with advanced squamous cell lung cancer. It might be worthy to conduct further researches to investigate the efficacy and safety of the combination therapy in such patients.

## 1 Introduction

Non-small cell lung cancer (NSCLC) represents the most commonly diagnosed carcinoma all over the world, which accounts for leading morbidity and mortality among all cancer types.^[1]^ Systemic therapy has been recommended as standard strategy for patients diagnosed with metastatic NSCLC. According to the recommendations from National Comprehensive Cancer Network (NCCN) guidelines, all patients diagnosed with adenocarcinoma, and selected patients with squamous lung cancer should receive driver gene test for detecing relevant mutations, including epidermal growth factor receptor (EGFR) mutations, anaplastic lymphoma kinase (ALK) rearrangements, ROS proto-oncogene 1 (ROS1) rearrangements, and V-raf murine sarcoma viral oncogene homologB1 (BRAF) V600E mutations, as well as expression level of the protein programmed death ligand 1 (PD-L1). Approximately 30% of patients with NSCLC harbor sensitive mutations, which might respond to targeted treatment or immunotherapy agents. Targeted therapy for sensitive mutations significantly improves progression-free survival (PFS) and overall survival (OS), along with fewer incidences of toxicities compared to cytotoxic chemotherapy. For patients without sensitive alterations, immune checkpoint inhibitors (ICI) as monotherapy or in combination with chemotherapy are superior to chemotherapy alone, which improves the OS.^[2,3]^ However, there are few effective strategies for patients with squamous cell lung cancer type, in which the sensitive alterations rate is less than 5%. In addition, antiangiogenic agent of bevacizumab, is not approved by Food and Drug Administration (FDA) in patients with advanced squamous cell lung cancer because of potential toxicities such as bleeding.^[4]^ It is urgent to seek effective and promising strategy during the management of patients with advanced/metastatic squamous cell lung cancer.

Apatinib (Hengrui Co. Ltd, Jiangsu, China), a novel tyrosine kinase inhibitor, selectively inhibits vascular endothelial growth factor receptor-2 (VEGF-2), blocks VEGF-mediated endothelial cell migration, and suppresses vessel formation. It is approved as further line of treatment in patients with chemotherapy-refractory advanced metastatic gastric cancer in China, based on its encouraging results in phase II and III clinical trials.^[5,6]^ Nevertheless, results from a phase II, placebo controlled research also suggested that apatinib significantly prolongs the PFS compared to placebo (4.7 vs 1.9 months, *P* < .001) in patients with advanced non-squamous NSCLC.^[7]^ However, limited literature supported apatinib as salvage treatment in patients with advanced squamous cell lung cancer. S-1, an oral fluoropyrimidine, of which combinative formulation of three pharmacological compounds, including tegafur, gimeracil, and oteracil potassium, at a molar ratio of 1:0.4:1, has become an alternative agent and widely used among Asian patients with advanced or metastatic gastric cancer,^[8]^ breast cancer,^[9]^ and pancreatic carcinoma.^[10]^ Several clinical researches have been conducted to assess the efficacy and safety profile of S-1 in patients with advanced squamous cell lung cancer,^[11,12]^ results of which revealed that maintenance S-1 might be an effective and feasible treatment for patients with advanced squamous cell lung cancer. However, the attempts were confined to the maintenance therapy with mono-S-1 in chemotherapy-naive patients. Few researches have been performed to evaluate the efficacy of S-1 as salvage therapy for patients with advanced squamous cell lung cancer.

Herein, we present a case of heavily pretreated advanced squamous NSCLC who responded to a regimen of low-dose apatinib combined with S-1 treatment, and achieved a long-term PFS as 24 months, and still in extension. We hope the case report would provide a new insight into the salvage treatment of advanced squamous cell lung cancer in clinical practice.

## 2 Case presentation

**Figure 1:**
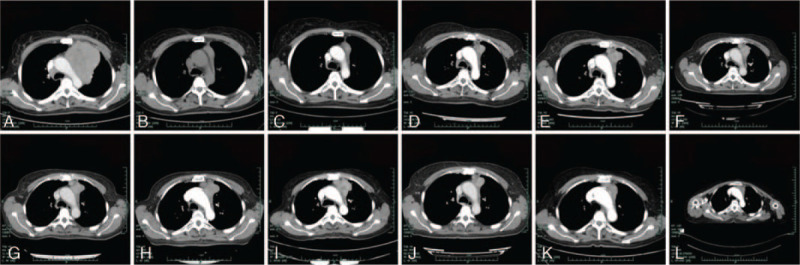
Chest CT scan showing the mass on the left upper lobe. A. Nov. 14 2014, B. May 5 2015, C. Oct. 28 2015, D. Dec. 20 2015, E. Sep. 28 2016, F. Jan. 23 2017, G. Mar. 11 2017, H. June 8 2017, I. Nov. 13 2017, J. Sep. 7 2018, K. Dec. 4 2018, L. Apr. 6 2019.

**Figure 2:**
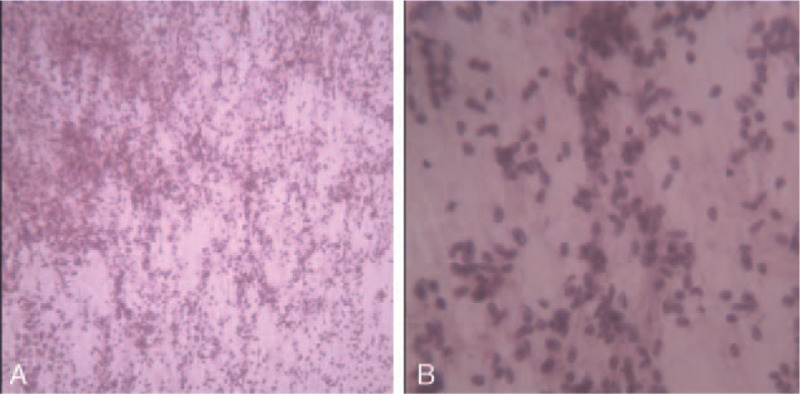
Histological findings of tumor tissue. A. × 40, B. × 200.

A 49-year-old female patient was referred to our hospital on October 21, 2014 due to dry cough for 1 month without any relief by medication. Besides, she denied smoking or any other medical or family history. A chest computed tomography (CT) scan revealed a lesion sized 80 × 41 mm in the left upper lobe, along with enlargement of bilateral supraclavicular lymph nodes (Fig. 1A). Otherwise, results from abdominal CT and cranial magnetic resonance imaging (MRI) did not suggest any suspicious lesions. Finally, pathologic findings from percutaneous lung lesion biopsy revealed squamous cell lung cancer with poor-differentiation (Fig. 2). Drive gene of EGFR could not be performed because of the potentially low incidence of available mutations for squamous cell lung cancer. Based on these, the patient was clinically diagnosed as lung squamous cell lung cancer on the left upper lobe, along with enlargement of bilateral supraclavicular lymph nodes suggesting metastasis, which was staged as IIIb (AJCC version 7).

**Figure 3:**
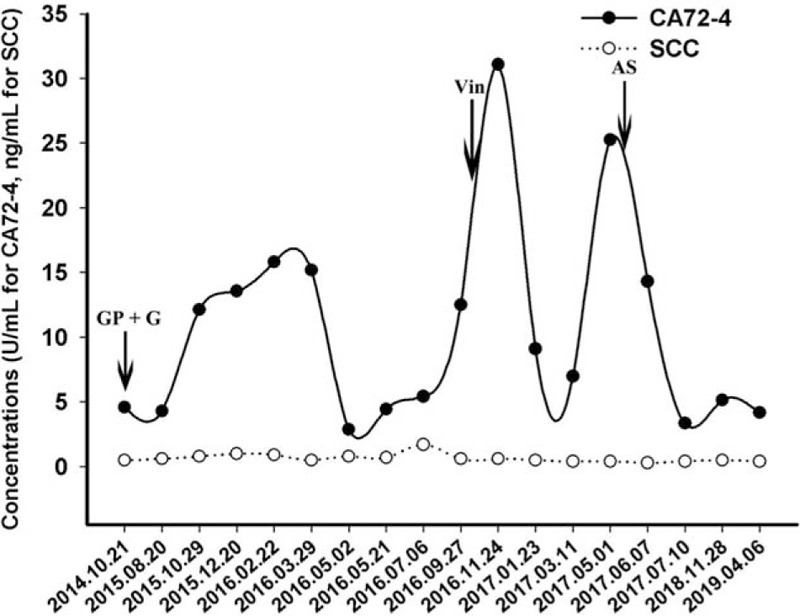
The changes of tumor markers including CA72–4 and SCC for each visit from the initial treatment to the present. GP + G: gemcitabine plus cisplatin as first-line treatment followed by gemcitabine alone as maintenance therapy; Vin: vinorelbine alone as second line treatment; AS: apatinib plus S-1 as third line treatment.

After the definite diagnosis, the patient received 6 cycles of platinum-based chemotherapy (gemcitabine 1000 mg/m^2^ day 1 and 8 plus cisplatin 75 mg/m^2^ day 1, every 21 days) in another hospital from November 19, 2014. The efficacy assessment with chest CT was evaluated as partial response (PR), according to criteria of RECIST version 1.1 (Fig. 1B). During this period, the suggested concurrent radiotherapy was refused by the patient herself because of the poor economic status. After the completion of first-line chemotherapy, the patient received maintenance therapy with single gemcitabine (gemcitabine 800 to 1000 mg/m^2^ day 1 and 8, every 21 to 28 days, depending on the recovery of neutropenia at grade 1 to 2 according to the criteria of Common Terminology Criteria for Adverse Events version 4.0) from April, 2015 to July, 2016. Regular image review of chest CT suggested sustained status of PR (Fig. 1B–D, RECIST version 1.1). Disease progression occurred after 15 months of maintenance therapy on September 28, 2016, Chest CT revealed an enlargement of the tumor on the left upper lobe (Fig. 1E). Due to the progression of disease, mono-therapy with vinorelbine (vinorelbine 25 mg/m^2^ day 1 and 8, every 21 days) was adopted as second-line treatment. The second line treatment lasted for four cycles, resulting in another 9 months of PFS. On June 8, 2017, the patient was referred to our outpatient again for emerging pain in left back and shoulder. Repeated chest CT showed an enlargement on the left upper lobe again (Fig. 1H), with metastasis on second thoracic vertebrae and rib. To control the cancer related pain, the patient received oxycodone at 10 mg every 12 hours. At this time, she had an Eastern Cooperative Oncology Group performance status (ECOG) of 2. Because of the long term venous infusion with chemotherapeutics drugs received, she refused to receive any further intravenous chemotherapy. Thus, we suggested her to receive apatinib at a dose of 250 mg per day as salvage therapy. In consideration of the significantly low dose of the apatinib treatment (apatinib at 850 mg per day in salvage therapy in gastric cancer during phase III research), we prescribed the addition of oral S-1 (50 mg twice per day, day 1 to 14, every 21 days) as combined treatment to inhibit the progression of cancer. As a result, the subsequent chest CT revealed continuous disease stable (according to RECIST version 1.1) from then on (Fig. 1I–L). The variations in size of the target tumor on the left upper lobe of lung are presented in the Table 1. She denied any other antitumor therapy including herbs. The regimen was still effective in the last follow-up on April 6, 2019 (Fig. 1L). The PFS for the administration of apatinib combined with S-1 has already up to 24 months and still in extension. The patient felt well with the painkiller oxycodone at 20 mg every 12 hours at the time of last follow-up on April 6^th^ 2019. No evidence of progression or metastasis was observed. In addition, no obvious toxicities including hypertension or hand-foot syndrome was observed during the management with apatinib combined with S-1. The tumor markers including CEA, CA125, CA153, CA19–9 were in the normal range during the whole treatment period. Tumor markers of CA72–4 and squamous cell carcinoma antigen (SCC) were slightly higher than the normal range, results of which are presented in the Figure 3. To specify potential sensitive mutations, we did the supplemental drive genes detectionusing plasma sample with next-generation sequencing (NGS) panel including anaplastic lymphoma kinase (ALK), ROS proto-oncogene 1 (ROS1), V-Ki-ras2 Kirsten rat sarcoma viral oncogene homolog (KRAS), neuroblastoma RAS viral oncogene homolog (NRAS), RET proto-oncogene (RET), V-raf murine sarcoma viral oncogene homologB1 (BRAF), receptor tyrosine-protein kinase erbB-2 (ERBB2), RAC-alpha serine/threonine-protein kinase (AKT1), discoidin domain receptor tyrosine kinase 2 (DDR2), fibroblast growth factor receptor 1 (FGFR1), MNNG HOS transforming gene (MET), phosphatase and tensin homolog (PTEN), phosphatidylinosito-4,5-bisphosphate 3-kinase (PIK3CA), and mitogen-activated protein kinase 1 (MAP2K1), result of which showed as wild types.

**Table 1:** Changes in size of the target tumor.

Date	Size of the target tumor (LD × SD)
Nov. 14 2014	8.0 cm × 4.1 cm
May 5 2015	5.5 cm × 1.8 cm
Oct. 28 2015	4.2 cm × 1.3 cm
Dec. 20 2015	2.2 cm × 1.8 cm
Sep. 28 2016	3.6 cm × 3.0 cm
Jan. 23 2017	2.9 cm × 2.7 cm
Mar. 11 2017	3.3 cm × 2.6 cm
June 8 2017	4.6 cm × 3.5 cm
Nov. 13 2017	4.5 cm × 4.3 cm
Sep. 7 2018	4.1 cm × 3.3 cm
Dec. 4 2018	3.2 cm × 1.7 cm
Apr. 6 2019	2.6 cm × 1.3 cm

LD = longest diameter, SD = shortest diameter.

## 3 Discussion

To our knowledge, the present case is the first one to report a patient with heavily pretreated advanced squamous cell lung cancer who achieved a long-term response to the combination therapy with low-dose apatinib and S-1.

Squamous cell lung cancer is one of the most common pathological types in patients with NSCLC.^[13]^ Though a few patients diagnosed with squamous cell lung cancer harboring sensitive mutations might benefit from the administration of tyrosine kinase inhibitors, the efficacy is still inconclusive. In addition, few strategies have been proven effective as further line treatment in patients with advanced squamous cell lung cancer.

Angiogenesis plays a significant part in the growth and metastasis of NSCLC. Bevacizumab, a humanized monoclonal antibody against VEGF, is recommended as the standard therapy for patients with advanced or metastatic lung adenocarcinoma according to NCCN Guidelines. However, the drug is not approved for the treatment of squamous cell lung cancer because of the potential adverse events including pneumorrhagia and pulmonary embolism.^[4]^ In addition, as an anti-VEGFR2 inhibitor, ramucirumab was also attempted for the salvage therapy in patients with NSCLC.^[14]^ However, the results of subgroup analysis revealed that regimen of ramucirumab combined with docetaxel did not significantly prolong survival time compared to docetaxel alone in patients with squamous cell lung cancer (9.5 versus 8.2 months, HR 0.88, 95%CI: 0.69 to 1.13). Nevertheless, more than 70% of patients suffered grade 3 or higher adverse events in both groups (79% for ramucirumab/docetaxel versus 71% for docetaxel alone). As another emerging agent of angiogenesis in China, apatinib has been approved as salvage treatment in patients with gastro-esophageal adenocarcinoma or advanced gastric adenocarcinoma as third-line or further lines choice.^[5,6]^ Apatinib is widely attempted for the salvage therapy in multiple types of advanced/metastatic solid carcinomas including hepatocellular carcinoma,^[15]^ breast cancer,^[16]^ colorectal cancer,^[17]^ and NSCLC.^[18]^ However, reducing the dose of apatinib is adopted in majority of researches because of the potentially severe adverse events. In the present case, a dose of 250 mg per day of apatinib was administrated for the present patient. As a result, there was no obvious adverse event observed including hypertension, proteinuria, hand-foot syndrome, diarrhea, or neutropenia during the whole treatment period.

S-1 is an oral fluoropyrimidine, which is widely used in patients with advanced or metastatic disease in Asian. It is reported that S-1 combined with carboplatin might be effective as first line treatment in patients with advanced NSCLC regardless of tumor histology.^[19]^ More recently, in a subset analysis of LETS study, patients with squamous cell carcinoma experienced a longer median survival in the carboplatin plus S-1 group than in the carboplatin plus paclitaxel group (14.0 versus 10.6 months, respectively), suggesting that S-1 may be one of the effective agents for squamous cell carcinoma.^[12]^ Besides, the application of regimen of low dose of apatinib combined with S-1 in the present case was also inspired from the clinical trial AEROC.^[20]^ In that research, the combination of apatinib with oral etoposide showed promising efficacy and manageable toxicities in patients with platinum-resistant or platinum-refractory ovarian cancer. Based on those evidence and clinical experience, the combination of low dose apatinib and S-1 was administrated as salvage therapy in the present patient. As a result, the patient obtained a satisfactory PFS. Hence the combination of low dose of apatinib and S-1 might be effective, with manageable toxicities in pretreated patients with advanced squamous cell lung cancer; however, this needs further investigation in a larger population.

In summary, we report a pretreated patient with advanced squamous cell lung cancer, who achieved a long term response to salvage therapy with low dose apatinib combined with S-1. The superior efficacy and manageable toxicities were satisfactory. However, the potential mechanism remains unclear. Further research is needed to confirm the efficacy, and investigate the potential mechanism of this combination treatment in pre-treated patients with advanced squamous cell lung cancer.

## Acknowledgments

The authors thank the patient for her participation and her agreement to publication of the report.

## Author contributions

**Conceptualization:** Jianxin Chen.

**Data curation:** Jianxin Chen.

**Investigation:** Yan Zou.

**Methodology:** Junhui Wang.

**Resources:** Junhui Wang.

**Validation:** Jianxin Chen.

**Writing – original draft:** Jianxin Chen.

**Writing – review & editing:** Yan Zou.
